# Healthcare seeking for diarrhoea, malaria and pneumonia among children in four poor rural districts in Sierra Leone in the context of free health care: results of a cross-sectional survey

**DOI:** 10.1186/1471-2458-13-157

**Published:** 2013-02-20

**Authors:** Theresa Diaz, Asha S George, Sowmya R Rao, Peter S Bangura, John B Baimba, Shannon A McMahon, Augustin Kabano

**Affiliations:** 1 Knowledge Management and Implementation Research Unit, Health Section, Programme Division, United Nations Children’s Fund (UNICEF), NY, New York, USA; 2 Department of International Health, Bloomberg School of Public Health, John Hopkins University, Baltimore, MD, USA; 3 Statisticians without Borders, American Statistical Society, Department of Quantitative Health Sciences, University of Massachusetts Medical School, MA, Worcester, USA; 4 Center for Health Quality, Outcomes, and Economics Research, Bedford VA Medical Center, Bedford, MA, USA; 5 Statistics Sierra Leone, Freetown, Sierra Leone; 6 United Nations Children Fund (UNICEF), Freetown, Sierra Leone

## Abstract

**Background:**

To plan for a community case management (CCM) program after the implementation of the Free Health Care Initiative (FHCI), we assessed health care seeking for children with diarrhoea, malaria and pneumonia in 4 poor rural districts in Sierra Leone.

**Methods:**

In July 2010 we undertook a cross-sectional household cluster survey and qualitative research. Caregivers of children under five years of age were interviewed about healthcare seeking. We evaluated the association of various factors with not seeking health care by obtaining adjusted odds ratios and 95% confidence limits using a multivariable logistic regression model. Focus groups and in-depth interviews of young mothers, fathers and older caregivers in 12 villages explored household recognition and response to child morbidity.

**Results:**

The response rate was 93% (n=5951). Over 85% of children were brought for care for all conditions. However, 10.8% of those with diarrhoea, 36.5% of those with presumed pneumonia and 41.0% of those with fever did not receive recommended treatment. In the multivariable models, use of traditional treatments was significantly associated with not seeking outside care for all three conditions. Qualitative data showed that traditional treatments were used due to preferences for locally available treatments and barriers to facility care that remain even after FHCI.

**Conclusion:**

We found high healthcare seeking rates soon after the FHCI; however, many children do not receive recommended treatment, and some are given traditional treatment instead of seeking outside care. Facility care needs to be improved and the CCM program should target those few children still not accessing care.

## Background

Sierra Leone emerged in 2002 from 11 years of civil war, and today has some of the worst health indicators in the world. In 2010, infant mortality was estimated to be 114 per 1000 live births and under five mortality was estimated to be 174 per 1000 live births, ranking Sierra Leone fifth highest in the world for child mortality [1]. In 2008, Sierra Leone only had 1.9 physicians, nurses and midwives per 10,000 populations in contrast to the estimated 23 per 10,000 population estimated to be needed to deliver basic maternal and child health care services [2]. Government primary health care services consists of peripheral health units, with the latter staffed by nurses, paid community health officers and maternal and child health officers. Villages are also served by travelling drug peddlers and a range of herbalists.

In 2005, due to the high child mortality and the severe lack of health manpower, the Ministry of Health and Sanitation (MOHS) of Sierra Leone allowed non-governmental organizations to deliver community case management (CCM) with community health volunteers (CHVs) in rural areas to increase coverage of treatment for malaria (artemisinin-based combination therapies [ACT]), pneumonia (co-trimoxazole), and diarrhoea (oral rehydration solution [ORS]) for sick children. This form of CCM was first piloted in Kono district in 2006. To further pilot this approach it was decided to extend this type of CCM with the addition of zinc for diarrhoea to two additional poor and marginalized districts. Selection of additional districts was based on a composite score that included wealth status, immunization and stunting rates and availability of health services. Of the 7 districts with the worst composite score, 6 did not have CCM. Of these 6, 2 were chosen in two different parts of the country (Kambia, Pujehun) to receive CCM and 2 remaining districts nearby that would not receive CCM were chosen as a comparison (Kailahun and Tonkolili). In addition to piloting CCM, in April 2010, the government of Sierra Leone launched the Free Health Care Initiative (FHCI) for pregnant and breast-feeding women and children under five years of age. The removal of user fees for this target group was also supported by a strengthening of the drug supply chain and increased pay for health workers. In the month before FHCI began, about 170,000 children received care from Sierra Leone’s hospital facilities, while in the month after FHCI, the number exceeded 340,000 [3].

Although it has been shown that CCM is very effective and CHWs can supplement facility services in rural underserved areas, [4-7] it is not known how such a program should be implemented in the context of removal of user fees in public health facilities. We describe the results of baseline quantitative and qualitative assessments conducted after the implementation of the FHIC and prior to implementation of CCM, relating to child health care seeking for diarrhoea, pneumonia and fever in children less than five years of age in four districts in Sierra Leone. Our research was conducted for the purpose of understanding health use patterns and as the baseline data collection for evaluation for CCM. We examined the initial responses of caregivers to the FHCI, gathered some information on the type of care being delivered through this initiative to better inform the implementation of the CCM program.

## Methods

### Sampling for quantitative household survey

Between June and July 2010 a household cluster survey was conducted in four districts (two districts that would receive CCM in the future and two comparison districts that would not receive CCM) to obtain baseline data before the implementation of CCM. A sample of 1500 households in each of the four districts was selected for the survey (3000 for the districts that would receive CCM and 3000 for the districts that would not receive CCM). Within each district, 50 clusters were selected based on population proportional to size sampling. Using personal digital assistants (PDA) with global positioning system (GPS) devices, household enumeration was conducted in each cluster and 30 households were randomly sampled. Details of this method have been described elsewhere [8].

### Quantitative household survey questionnaires

Three questionnaires were used based on previous Demographic and Health Survey (DHS) and Multiple Indicator Cluster Survey (MICS) questionnaires for each household. The head of the household or a responsible adult was interviewed to determine household characteristics, and caregivers of all children under five years of age were asked about child morbidity and care seeking for diarrhoea, respiratory and fever symptoms two weeks prior to the survey. The questionnaire was programmed using Visual CE (Cambridge, MA) professional edition version 12 with multiple data checks and uploaded onto PDAs.

Heads of households in Sierra Leone were asked permission to interview others in the household including girls 15 to 17 years of age, prior to seeking consent from all women/caretakers over 18 years of age and girls under 18 who are married or have children. Laminated consent forms were provided to all interviewers. Given the high illiteracy rate verbal consent was obtained. The interviewer was required to check off on the PDA whether the consent was read and whether permission was given to be allowed to proceed with the interview.

For those children who were ill in the recall period, caregivers were asked whether or not they had sought treatment for the illness from any source. If they had, they were asked where they sought treatment first (e.g. public hospital, community health center, mobile clinic, pharmacy, private hospital or physician, another house); from whom (e.g. physician, nurse, health aide, pharmacist, paid community health officer, peddler, CHV, herbalist); and with visual aids, what type of treatment they received (e.g. ORS, zinc, co-trimoxazole, ACT, other anti-malarials, another antibiotic, another pill that is commonly used to control fever or diarrhoea but was not an antibiotic). Caregivers were also asked if they had used any other remedy including homemade sugar salt solution (SSS), traditional remedies such as drinking hot fluids with herbs, hot sponging with goat fat soap, goat oil ointment, and boiled herbs.

### Analysis of quantitative household survey

Our analysis is only based on children under 5 whose caretakers were interviewed. We computed weights that accounted for the complex survey design and non-response and used them in all our analyses that were conducted in SAS version 9.1 [9]. We calculated wealth quintiles within our sample based on household assets using principle components analysis [10]. Two week prevalence of diarrhoea and fever was calculated as the proportion of all children with such symptoms. In addition ‘possible pneumonia’ was defined as cough with or without fever and difficulty breathing that was due to a problem in the chest; the standard definition used in DHS and MICS surveys. Recommended treatment was defined as follows: for diarrhoea, zinc and ORS (but not homemade SSS); for pneumonia, any antibiotic; and for fever, ACTs. To calculate distance from the household to the nearest clinic we compared our GPS data to a national GPS survey of health facilities conducted in 2010. We calculated straight line distances to the nearest health facility.

We present weighted percentages and 95% confidence limits for description of our sample. To test for associations of the factors with not seeking health care in the bivariate analysis we used 2-sided chi-square tests; p values <0.05 were considered significant. Odds ratios (OR) and 95% confidence intervals (95% CI) were obtained from a multivariable logistic regression model to assess the factors associated with not seeking health care that controlled for all variables associated with not seeking care in the bivariate analysis or were reported in the literature (age of child, educational level of mother, distance from health facility) as being associated and had conceptual reasons for inclusion.

### Qualitative survey

The qualitative study aimed to provide formative information for the baseline survey and to obtain in-depth information on household recognition and response to child morbidity, particularly along the following themes: illness concepts, terms, causation, prevention, treatment, decision making, barriers to care, patterns of resort, provider preferences, and care burdens. The study design was informed by applied qualitative research, [11,12] an approach that focuses on specific illnesses (diarrhoea, pneumonia, malaria), addresses programmatic concerns related to these illnesses (identifying local causes, terminology and treatment for diarrhoea), and draws from experiences of actual cases of sick children. Focus group discussions captured social norms as expressed by specific sub-groups of concern, while in-depth interviews collected narratives of actual cases of care seeking for child illness.

Data collection took place in two stages, first in April 2010 and later in July 2010, by data collectors who received 5 days of training on qualitative methods and research ethics and were accompanied in the field by a qualitative research supervisor. Data collection focused on three types of villages: those with a community health post, those with a community health center, and those without any government facility in the village and typically located 3–10 miles away or five hours distance from a village with a government health facility.

Focus group discussions and in-depth interviews were conducted with mothers, fathers and older caregivers of children under age five. Participants were selected purposively with the intent to maximize information on child illness and care seeking by drawing on mothers, fathers and caregivers of children under five years of age with the aid of key informants including village elders and health volunteers with efforts made to include a diversity of respondents among in-depth interviews including those living near to and far from the village center. Informed, verbal consent was sought and received from all respondents who partook in 36 focus group discussions and 64 in-depth interviews, including 15 follow-up interviews, which were completed and taped across 12 villages in all 4 districts. Debriefing sessions based on field notes were held during and immediately after fieldwork to further explore deviant or unique findings that merited follow up interviews and to support reflective practice and possible differences in interviewing, probing and interpretation among data collectors. One respondent declined consent due to time constraints. Data collection ceased upon saturation of key themes listed earlier.

Data were transcribed into English and a list of hierarchical codes developed and validated by a co-investigator before being applied to the dataset using Atlas version 4.1 [13]. Thematic analysis was undertaken that compared and contrasted data from different respondents, data collection methods and sites to arrive at triangulated descriptions of illness terminologies, causation, prevention, and treatment patterns. Data summaries were shared with key stakeholders before developing manuscripts. The findings presented here are those that help to further elucidate quantitative findings.

The study received institutional review board approval from the Government of Sierra Leone Office of Science and Ethics Review Committee, Ministry of Health.

## Results

Of 6000 households approached for interview 5527 (92.1%) agreed to participate. Of these households 67% had children under 5 for a total of 6429 children of which 5966 (92.8%) completed questionnaires. Of these 15 were removed due to missing data or incorrect birthdates resulting in 5951 children. Response rates did not differ between districts that would implement CCM and those that would not. Non-response was due mostly to lack of availability of the appropriate respondent. Overall 31.3% of households with children were polygamous, the vast majority were Muslim (85.6%) and most were from Mende (36%) or Temne (45.3%) tribes (Table 1).

**Table 1:** Characteristics of households and children, baseline community case management survey (CCM) in 4 districts in Sierra Leone 2010

	**Districts that will receive CCM**		**Districts that will not receive CCM**		**Total**	
	**N= 3152**		**N=2799**		**N = 5951**	
**Variable**	**Weighted%**	**95% C.L.**	**Weighted%**	**95% C.L.**	**Weighted%**	**95% C.L.**
**Polygamous household**	38.5	33.8-.66.2	25.4	18.2-32.7	31.3	26.9-35.7
**Religion of head of household**						
Islam	94.9	92.9-96.9	77.9	72.0-83.8	85.6	82.1-89.0
Christian	4.9	2.9-6.9	21.9	16.1-27.8	14.2	10.8-17.7
Other	0.2	00--0.6	0.2	0-0.3	0.2	0.0-0.4
**Tribe of household:**						
Mende	43,4	31.7-55.0	29.9	19.1-40.8	36.0	27.9-44.1
Temne	33.7	23.9-43.5	54.8	42.8-66.9	45.3	36.9-53.7
Susa	12.1	6.1-18.1	0.1	0.0-.0.2	5.5	2.6-8.4
Limba	8.1	3.5-12.7	4.4	0.7-8.1	6.0	3.1-8.9
Kissi	0.0	3.5-12.7	5.6	2.4-8.7	3.1	1.4-4.8
Koranko	0.0	0.0-0.0	3.9	0.0-7.9	2.1	0.0-4.3
Other	3.6	0.0-0.1 0.9-4.5	1.3	0.5-2.1	2.0	0.3-2.9
**Selected assets of household:**						
Radio	43.6	38.1-49.2	35.2	30.9-39.4	38.9	35.5-42.5
Mobile phone	30.6	26.7-34.4	23.3	18.9-27.7	26.6	23.6-29.6
No sanitation	33.3	26.7-38.8	38.7	31.9-45.4	36.3	31.5-41.1
Unprotected water	53.4	44.6-62.2	61.5	41.6-61.6	57.9	51.4-64.4
Dirt floor	78.2	74.0-82.3	80.6	76.9-84.4	79.5	76.8-82.3
Electricity	1.7	0.7-2.8	0.3	0.1-0.5	0.9	0.4-1.5
Wood cooking	98.7	97.6-99.7	98.9	97.8-100	98.8	98.0--99.6
Atleast one women in household went to school	26.7	39.1-49.8	25.8	21.8-29.8	26.5	39.1-49.8
Proportion of Households in poorest Wealth Quintile:	18.7	14.1-23.3	24.8	20.1-29.4	22.0	18.7-25.4
Household distance to nearest health facility:						
< 5 Km	77.5	67.1-87.9	59.2	46.5-71.9	67.4	58.6-76.3
> 5 Km	20.6	11.1-30.0	25.0	12.4-37.6	23.0	14.8-31.2
unknown	2.0	0.4-3.5	15.8	6.4-25.1	9.6	4.2-14.9
Male gender of child	50.0	47.5-52.4	48.9	46.8-51.0	49.4	47.8-51.0
If child is infant	24.8	22.1-27.6	24.7	23.0-26.6	24.8	23.2-26.4
2 week prevalence::						
Diarrhoea	26.5	23.2-29.7	24.9	21.4–28.5	25.6	23.2-28.0
Fever	67.1	62.8-71.4	65.5	62.1-68.9	66.3	63.6-69.0
Possible pneumonia	16.9	12.2-21.6	22.0	19.4-24.7	19.7	17.2-22.2

**Figure 1:**
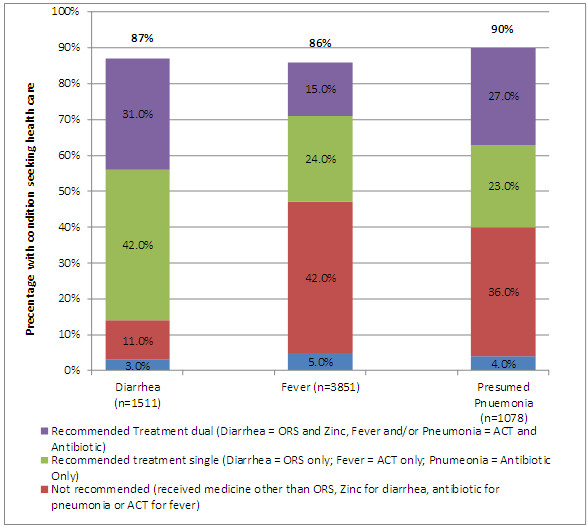
Health seeking behaviors and type of treatment by condition, baseline community case management survey in 4 districts, Sierra Leone 2010**.

Over half of the children (66.2%) had fever, 25.6% had diarrhoea and 16.9% had pneumonia in the two weeks prior to the interview. These proportions were similar between CCM districts and comparison districts (Table 1). Multiple conditions were common. Overall, 94% of children with presumed pneumonia and 84% of children with diarrhoea had fever, and 23% of children with diarrhoea had presumed pneumonia. Healthcare seeking behaviours were very high; over 85% of children were brought for care for all conditions. Healthcare seeking was highest for pneumonia with 90% of caretakers seeking outside care. Overall, among those that sought treatment, the proportion that did not receive recommended treatment was highest for fever (41%) followed by presumed pneumonia (36.5%) and lowest for diarrhoea (10.8%) (Figure 1).

**Figure 2:**
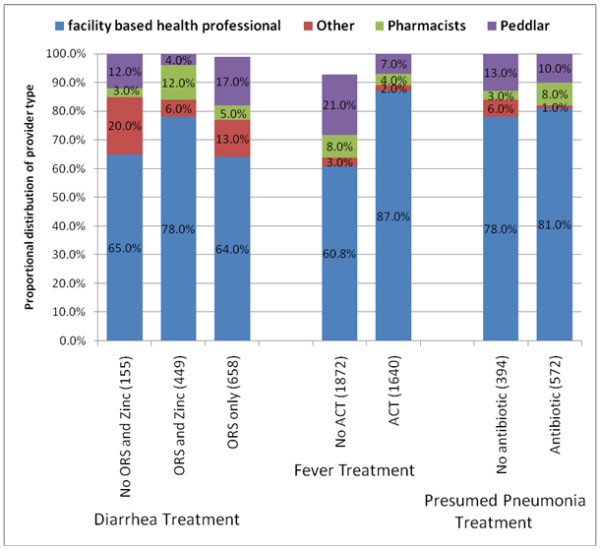
Proportional distribution by provider type of receipt of recommended treatment* or not, community case management baseline survey in 4 districts, Sierra Leone 2010.

Among those that sought care outside the home, over 75% of children were brought to a public facility, regardless of their illness type. The most common provider of treatment (60% or higher) were facility based health professionals (e.g., doctors, nurses, health aides and community health officers), regardless of whether the treatment was recommended or not (Figure 2). Among providers other than facility based health professionals, there were notable differences between those giving recommended vs. not recommended treatments. The proportion of children with diarrhoea that received ORS and Zinc from providers other than facility based health professionals was 12% for recommended treatments but 30% for not recommended treatments. The proportion of children with fever received ACT from a peddler was 7% but 21% among those that did not receive ACT. The proportion of children that received an antibiotic for presumed pneumonia from others was 1% but 6% among those that did not receive an antibiotic (Figure 2).

In the multivariable model group was not significantly associated with not seeking care for diarrhoea and fever but for presumed pneumonia the group receiving CCM was more likely to not seek care (AOR 1.8, 95% CI 1.0 -3.3) (Table 2). For children with diarrhoea those who were given homemade SSS were more likely to not seek healthcare (AOR 2.0, 95% CI: 1.1-2.4). Also among children with diarrhoea those who had one of the other two conditions were less likely to not seek healthcare AOR=0.8; 95% CI: 0.6-1.1); children who had all three conditions were the least likely to not seek treatment for diarrhoea (AOR=0.6; 95% CI: 0.3-1.2). Use of traditional treatments was the one variable significantly associated with not seeking care for all three conditions with AORs ranging from 1.5 to 3.5 (Table 2). Social determinants (polygamy, religion, wealth quintile, mother’s education, and distance from health facility) were not associated with health care seeking outside the home for children with diarrhoea and fever. However, for pneumonia children who were from the lowest quintile were less likely to not seek care than others (AOR 0.5, 95% C.I. 0.3-0.8),

**Table 2:** Factors associated with not seeking treatment outside the home, by disease, baseline community case management (CCM) survey in 4 districts in Sierra Leone 2010*

		**Bivariate**				**Multivariable logistic regression model**		
**Variable**	**N**	**% not seeking healthcare**	**Odds**	**95% confidence interval**	**P value**	**Adjusted odds ratio**	**95% confidence interval**	**P value**
**Diarrhea**	1511							
**District type:**								
Will not receive CCM Will receive	719	10.2%						
CCM	792	16.5%	1.7	1.2,2.5	.0009	1.5	0.9,2.4	0.09
**Age:**								
≥1 year	1167	13.7%						
< 1 year	344	11.6%	0.8	0.7,2.1	0.5	0.7	0.4,1.2	0.2
**Gender:**								
Male	772	12.6%						
Female	739	13.9%	1.1	0.7,1.7	0.6	1.0	0.6,1.5	0.9
**Tribe of household:**								
Other	315	16.1%						
Temne	505	10.2%	0.6	0.3,1.1		0.6	0.3,1.5	
Mende	691	14.3%	0.9	0.5,1.4	0.2	1.0	0.6,1.8	0.5
**Religion:**								
Other	240	8.5%						
Muslim	1089	14.1%	1.7	0.9,3.3	0.08	1.6	0.7,3.8	0.2
**Polygamy**:								
No	1075	12.4%						
Yes	436	15.2%	1.3	0.8,1.9	0.2	1.3	0.9,1.9	0.2
**Household distance to nearest health facility:**								
< 5 Km	1071	12.4%						
≥ 5 Km		13.9%	1.2	0.4,1.5		1.0	0.3,3.0	
unknown	302	10.4%	0.8	0.3,1.7	0.6	1.0	0.4,2.7	1.0
	138							
**Wealth quintile:**								
Other	1132	13.5%						
lowest	379	12.3%	0.9	0.6,1.4	0.6	0.9	0.5,1.6	0.8
**Atleast one women in household went to school**								
No	1012	11.1%						
yes	347	13.5%	1.2	0.8,2.0	0.4	1.1	0.7,1.9	0.6
**Traditional treatment**								
No	1003	11.2%						
Yes	508	17.5%	1.7	1.2,2.5	.003	1.5	1.0,2.2	0.04
**Homemade SSS:**								
No	527	9.1%						
Yes	984	15.2%	2.0	1.2,2.5	.003	2.0	1.1,2.4	0.002
**Disease:**								
Diarrhea only	246	20.8%						
Also Fever or presumed pneumonia	909	12.9%	0.6	0.4,1.1		0.6	0.4,0.9	
Also fever and presumed pneumonia	356	8.8%	0.4	0.2,0.7	.0003	0.4	0.2,0.7	0.005
**Fever**	3851							
**District type:**								
Will not receive CCM Will receive	1845	13.0%						
CCM	2006	16.1%	1.3	1.0,1.7	.07	1.2	0.9,1.8	0.1
**Age:**								
≥1 year	2922	15.0%						
< 1 year	929	13.1%	1.2	0.9,1.6	0.3	0.9	0.6,1.3	0.6
**Gender:**								
Male	1904	14.5%						
Female	1947	14.5%	1.0	0.8,1.2	0.9	1.0	0.8,1.2	0.9
**Tribe of household:**								
Other	712	15.1%						
Temne	1287	14.9%	1.0	0.7,1.5		1.2	0.6,1.4	
Mende	1852	14.0%	0.9	0.7,1.2	0.8	1.1	0.8,1.7	0.7
**Religion**:								
Other	529	13.5%						
Muslim	3322	14.7%	1.1	0.8,1.7	0.6	0.9	0.6,1.4	0.8
**Polygamy:**								
No	2661	14.8%						
Yes	1190	14.1%	0.9	0.7,1.2	0.6	0.9	0.6,1.4	0.7
**Household distance to nearest health facility:**								
< 5 Km	2577	15.2%						
≥ 5 Km	968	12.1%	0.8	0.4,1.4		0.6	0.3,1.2	
unknown	306	15.7%	1.0	0.5,1.9	0.4	0.7	0.3,1.4	0.3
**Wealth quintile:**								
Other	2957	15.6%						
lowest	894	15.2%	1.0	0.5,1.9	0.2	0.7	0.5,1.0	0.08
**Atleast one women in household went to school**								
No	2611	13.6%						
yes	865	14.4%	1.1	0.8,1.4	0.6	1.1	0.8,1.4	0.5
**Traditional treatment**								
No	2963	11.6%						
Yes	888	24.7%	2.5	1.8,3.4	<0.001	2.9	2.1,4.0	<.0001
**Disease:**								
Fever only	1934	15.8%						
Also diarrhea or presumed pneumonia	1561	13.7%	0.7	0.4,1.2		0.8	0.6,1.1	
Also diarrhea and presumed pneumonia	356	11.3%	0.8	0.4,1.4	0.2	0.6	0.3,1.2	0.2
**Presumed Pneumonia**	1078							
**District type:**								
Will not receive CCM Will receive	607	8.7%						
CCM	408	12.5%	1.5	0.9,2.4	0.1	1.8	1.0,3.3	0.04
**Age:**								
≥1 year	838	9.8%						
< 1 year	240	11.4%	1.2	0.7,2.0	0.5	1.0	0.5,2.0	1.0
**Gender:**								
Male	524	9.6%						
Female	554	10.8%	1.1	0.7,2.0	0.6	0.9	0.5,1.7	0.8
**Tribe of household:**								
Other	188	8.8%						
Temne	262	12.3%	1.4	0.7,3.3		1.6	0.5,5.1	
Mende	628	9.9%	1.1	0.6,2.5	0.6	1.1	0.5,2.5	0.7
**Religion**:								
Other	155	7.3%						
Muslim	923	10.7%	1.5	0.8,3.1	0.2	1.3	0.5,3.4	0.5
**Polygamy:**								
No	732	9.2%						
Yes	346	12.1%	1.4	0.8,2.5	0.3	1.2	0.7,2.3	0.4
**Household distance to nearest health facility:**								
< 5 Km	798	11.0%						
≥ 5 Km	209	6.3%	0.4	0.1,1.4		0.4	0.1,1.4	
unknown	71	13.3%	1.2	0.4,3.4	0.2	0.5	0.1,1.9	0.3
**Wealth quintile:**								
Other	795	13.9%						
lowest	283	9.1%	0.6	0.3,1.0	0.05	0.5	0.3,0.8	0.008
**Atleast one women in household went to school**								
No	777	8.8%						
yes	183	14.6%	1.8	1.0,2.9	0.04	1.6	0.8,3.3	0.2
**Traditional treatment**								
No	776	7.1%						
Yes	302	18.4%	2.9	1.7,5.2	<.001	3.5	2.0,6.2	<.001
**Disease:**								
Presumed Pneumonia only	48	14.1%						
Also Fever or diarrhea	674	9.4%	0.8	0.3,2.2		0.6	0.2,1.8	
Also fever and diarrhea	356	11.3%	1.2	0.6,2.4	0.6	0.7	0.2,2.4	0.7

Qualitative data also documented references to high levels of health care seeking from a range of providers (village elders, herbalists, peddlers and government providers) for all three diseases, and at times a high level of confidence in care provided by personnel working in government facilities. For instance a mother in a remote village on discussing treatment for her child with presumed pneumonia reported the following:

R: As I arrived I reported myself to Nurse that my child is not well and she really noticed that the sickness was a serious one. She leaves all the work that she was doing to attend to us.

I: What did she do?

R: She gave the injection and also with some medicine, some to be given in the afternoon and others at night. And she also encouraged me that the child will get well with these medicine.

I: How did you see Nurse?

R: She is kind hearted. Nurse is always ready to help us day and night.

The qualitative data also highlighted reasons why respondents sought care from different kinds of providers depending on who they saw as working. Perceptions of receiving ineffective treatment due to symptoms not abating often led to respondents switching type of provider, as reported by young mothers from a remote village discussing care-seeking for malaria.

When that gbelui (malaria) starts you take her to the halay weibu (hospital) and you talk to older mothers that gbelui has catch your child and that you are taken her to the halay weibu and she is still sick so what next to (do?)? They will advise that I put herbs together to wash the child but this would depend if it will works for my child. So that is what I would stop: sometimes with the herbs, sometimes with the herbs and halay weibu.

While in some instances, respondents reported that the presence of government providers replaced peddlers, in other instances due to government providers not having medicines or facilities not existing, respondents turned to peddlers for treatment. For example, mothers in a remote village discussed resorting to traditional treatment for pneumonia due to lack of availability of government health facilities and lack of trust in drug peddlers:

M: why is it that it is only traditional medicine you are using to cure this sickness?

R6: since the hospital here was burned down unless we get traditional medicines and also those drug peddlers but even with them their drugs are not good.

Despite the lack of trust in drug peddlers expressed by some respondents, for those living in remote villages purchasing small quantities of drugs from a peddler was reported as more affordable and accessible, even if it did not mean a complete or appropriate regime of medication, than traveling across difficult terrain to access government providers.

Reasons for resorting to traditional treatments included both respect for village elders and herbalists as providers and causal beliefs that categorised the illness as not requiring biomedical treatment. At the same time, respondents also recounted resorting to traditional treatment due to lack of access to government services, lack of financial means or lack of trust in peddlers For example, fathers in a village with a government facility discussing treatment for diarrhoea and resort to elders for care due to lack of medicines from health facilities and trust in their care:

R: well the point is that if we do not have the medicine from the hospital, we do normally carry the child to the old mothers.

I: so you said, you carry the child to the old mother why?

R: the old mothers who knows the medicine can cure the child.

In terms of reporting barriers to care, despite the advent of FCHI, first and foremost was the expense of seeking care from government services, especially when located in remote villages. Fathers in a village situated with a government facility discussing treatment for malaria mentioning resort to traditional treatment due to costs in accessing care at health facilities:

R: Some times when our children get sick we don’t have money to take them to the hospital and we don’t want the illness to get worst so we treat them traditionally.

Lack of access to government facilities was also due to lack of facilities from destruction during the war, distance across difficult terrain, or due to the facility being closed when the child falls ill at night.

## Discussion

The results of our survey from four rural districts in Sierra Leone found higher rates of healthcare seeking behaviours for all three conditions (>85%) than have been reported in surveys prior to the FHCI. In the rural sample of the 2008 Demographic Health Survey (DHS), [14] 47% of children with diarrhoea, 45% of children with presumed pneumonia and 47% of children with fever were brought for health care. Similarly in the 2009 rural Sierra Leone District Health Services Baseline Survey (SLDHBS), [15] these rates were 40%, 50% and 52% respectively. In spite of this high rate of healthcare seeking behaviour many children did not receive the recommended treatment and there were still some children who were not brought to health care services, especially children that were given traditional treatments at home.

Our survey sample has similar demographic characteristics to the rural portion of past surveys, using the same sampling scheme, the same survey organization, and the same questions. However, we found a higher than expected prevalence for all three conditions. In the DHS [14] conducted in 2008 the two week prevalence rate of diarrhoea among the rural sample (similar to our sample) was 13.7%, for presumed pneumonia 7.4% and for fever 24.4% much lower than in our survey. Similarly, in the rural sample of the SLDHBS conducted in 2009 [15] the rates for diarrhoea were 10.5%, presumed pneumonia 5.2% and fever 21.2% again much lower than what we found. Our finding of higher levels of child morbidity than in previous surveys may be because we conducted the survey in the wet season while the other surveys were conducted in the dry season. During the wet season, illnesses, especially malaria, are usually higher. Additionally with the FHCI, respondents may be over-reporting illnesses in anticipation of receiving free services. The location of the questions within the survey and the skip patterns may have differed slightly from DHS and SLDHS which were much larger questionnaires, and we used a PDA, we do not know if these factors resulted in higher rates of reporting symptoms.

With regards to health care seeking, both quantitative survey and qualitative findings found higher levels than what was reported in surveys prior to FHCI. Our finding of higher healthcare seeking behaviours after FHCI is supported by data from the 2010 MICS survey conducted during the dry season 5 months after FHCI which found 74.3% of rural children with presumed pneumonia being brought to a health care provider [16]. These findings suggest that the FHCI resulted in an increased uptake of government health services. Increase in healthcare seeking after removal of user fees has been documented extensively in Africa [17-25]. These increases primarily benefit the poor [19,26].

Even though we found high healthcare seeking, a large proportion of children did not receive the recommended treatment they should have based on symptom reporting. Although receiving treatment from a peddler or someone other than government health personnel was more common among those not receiving recommended treatments, nurses, health aides, community health officers and doctors still provided the majority of not recommended treatments. In the qualitative study we found that the use of these alternative providers was in part due to unavailability of medicines at facilities and that families retained a critical perspective of providers, valuing those that provided effective treatment. Poor quality treatment and/or stock outs can undermine the potential impact of providing free health care. In Sierra Leone despite FHIC in some instances, drugs and other essential medical supplies was simply not available (Kabano A., UNICEF Sierra Leone, direct communication 2012) and there are claims that sometimes women were still charged for services [27]. In other countries abolition of user fees without proper planning did result in a decrease in overall quality of services, revenues and increases in difficulties meeting recurrent expenses such as purchasing medications [22,27-30].

Finally among the small proportion of ill children for whom families did not seek health care outside the home, those who used traditional treatments at home, were the most likely not to seek care. Qualitative data detailed households seeking traditional treatment due to preference and due to lack of alternatives. Delays in seeking treatment have been found to be associated with use of home treatments or self-medication in other countries in sub-Saharan Africa [25,31,32]. In addition, we found that children with diarrhoea who had multiple symptoms were more likely to be brought to a health care facility. Other studies in sub-Saharan Africa have also found that those with multiple symptoms were more likely to be brought to care [33,34].

With regards to social determinants, several studies have found an association between health care seeking and socioeconomic status as well as an association with mother’s level of education [33-36] but we did not find such an association except among children with presumed pneumonia where we found an opposite association than expected, children of lowest wealth quintile were actually more likely to seek healthcare. Perhaps the FHIC removed financial barriers to care, but we also must consider the fact that the population in our districts were uniformly poor so differences by wealth quintile may not be very large. Finally, in the household survey we did not find an association with distance from health facilities as has been found in other studies [31,33,35]. However, this lack of association may be because we only calculated a straight line distance, and did not take into account geographic obstacles (e.g. lack of roads, rivers, mountains) that could impact healthcare seeking. In the qualitative findings, respondents did report geographic obstacles as reasons for not seeking health care, particularly for those respondents living in more remote villages or that lived across roads and rivers that became impassable during the rainy seasons, living on islands, etc.

There are several further limitations to our research. Our survey was designed to inform and evaluate the CCM program that would be put in place and not the FHCI. It therefore did not include a pre and post FHCI survey design; however, other surveys conducted before and after the FHCI support our findings. We relied on self-reported symptoms and types of treatments; actual diagnosis of conditions may differ from reported symptoms and despite the use of pictures caretakers may not always accurately report what they received. We assumed inappropriate treatment based on symptoms but the actual physical exam may have revealed signs resulting in another diagnosis. As the qualitative research explored all three child morbidities of interest, respondent fatigue may have affected the quality of some responses.

## Conclusions

Our findings suggest increased healthcare seeking in the context of FHCI, but also point to the urgent need to improve facility level care to ensure proper diagnosis and treatment so as to avoid undermining the impact of providing free health care in all 4 districts surveyed. Our qualitative findings in particular highlight numerous barriers to care that still need to be addressed for child survival. For the 2 districts that will implement CCM the program should be designed to ensure proper diagnosis and treatment and target services to persons who still do not access care despite the FHIC.

## Abbreviations

ACT: Artemisinin-based combination therapies;CCM: Community case management;CHV: Community health volunteer;DHS: Demographic health survey;FHIC: Free health care initiative;MICS: Multiple indicator survey;ORS: Oral rehydration solution;PDA: Personal digital assistant;SLDHBS: Sierra Leone district health services baseline survey

## Competing interests

The authors declare they have no competing interests.

## Authors’ contributions

All authors contributed to the conception of the study. TD AG SM SR designed the study and analysed the data. PB was responsible for overseeing the data collection. All authors reviewed the data analysis and then provided interpretation of the findings. TD and AG drafted the manuscript and all other authors provided revisions. All authors have given final approval of the manuscript.

## Pre-publication history

The pre-publication history for this paper can be accessed here:

http://www.biomedcentral.com/1471-2458/13/157/prepub
